# Supplementary material for: Cardiovascular risk factors among nurses: A global systematic review and meta-analysis
Source: PLoS One. 2024 Mar 21;19(3):e0286245. doi: 10.1371/journal.pone.0286245 (PMC10956831; doi:10.1371/journal.pone.0286245)
Supplement: S1 File — (PDF) [file pone.0286245.s002.pdf]

First Author, Year	Year	Total sample	sample	shift work,ward	Total Male	Total Female	Country	Continent	WHO Regional classification	Age (Mean)	Age (SD)	Quality of Study	SBP ≥ 140mmHg	DBP ≥ 90mmHg	HDL<40	LDL>100	TC≥200	BMI(25–29.9)over weight	obese (BMI≥30)	current smoke	NO Physical inactivity	alcohol 3 or 4/wk	high Random blood sugar	history of CVD	Family history of CVD	%Waist circumference > 40 inches for men or > 35 inches for women
Solymanzadeh et al,2022	2022	120	60	Shift workers	26	34	Iran	Asia	EMRO	32.20	2.97	High	33	33	55	NG	12	NG	NG	5	NG	NG	NG	NG	NG	NG
		120	60	Shift workers	19	41				37.43	2.97		16	16	57	NG	4			3				NG	NG	NG
Buremoh et al,2020	2020	196	196	Shift workers	NG	NG	Nigeria	Africa	AFRO	39.3	7.4	High	3	NG	NG	NG	NG	80	80	6	45	18	3	13	91	114
Fair et al,2009	2009	1345	1345	Day workers	51	1294	United States	America	AMRO	47.37	8.7	High	21	NG	111	534	311	378	276	49	158	340	NG	23	275	NG
Burns et al,2010	2010	103	103	Shift workers	93	10	United States	America	AMRO	47.9	NG	Medium	18	18	10	NG	49	NG	NG	3	NG	NG	4	1	56	39
Silva et al,2017	2017	20	20	Shift workers	2	18	Brasil	America	AMRO	35.8	NG	Medium	5	4	NG	NG	NG	4	2	2	11	15	NG	NG	NG	NG
Faruque et al,2021	2021	938	938	Night workers	114	824	Bangladesh	Asia	SEARO	38.2	9.7	High	99	99	NG	NG	NG	371	90	10	863	9	182	NG	NG	NG
Gallagher et al,2017	2017	5041	2678	Shift workers	620	4421	Australia	Australia	WPRO	47.99	11.46	High	409	409	NG	NG	NG	NG	NG	497	NG	NG	193	NG	NG	NG
HA et al,2004	2004	226	226	Shift workers	0	226	Korea	Asia	SEARO	28.5	NG	High	5	5	NG	NG	7	NG	6	0	NG	10	0	NG	NG	NG
Zhao et al,2021	2021	84697	84697	Day workers	0	84697	China	Asia	WPRO	NG	NG	High	5095	5095	NG	5109	5109	NG	NG	269	18702	41674	872	NG	44695	NG
Yan et al,2021	2021	1344	1344	Shift workers	0	1344	China	Asia	WPRO	46.12	10.47	High	123	123	NG	NG	196	240	230	2	249	864	32	NG	460	NG
Martínez-Gurrión et al,2014	2014	195	195	Shift workers	9	186	Mexico	America	AMRO	39.5	8.5	Medium	75	75	NG	NG	NG	NG	NG	15	NG	NG	18	NG	113	NG
Monakali,2018	2018	203	203	Night workers	24	179	South Africa	Africa	AFRO	45.17	11.26	High	106	106	NG	NG	NG	NG	95	17	144	54	NG	NG	NG	NG
Nobahar et al,2015	2015	56	56	Night workers	6	50	Iran	Asia	EMRO	32.14	8.11	High	NG	NG	NG	NG	NG	15	6	1	22	NG	NG	NG	NG	NG
Donkor et al,2016	2016	95	95	Night workers	32	63	Liberia	Africa	AFRO	33.8	6.65	High	24	33	NG	NG	NG	47	28	NG	NG	NG	10	NG	NG	61
Riese et al,2000	2000	165	165	Night workers	NG	NG	Netherlands	Europe	EURO	33.7	8.1	High	NG	NG	NG	NG	NG	NG	NG	46	49	NG	NG	NG	NG	NG
Hammer et al,2022	2022	20701	20701	Day workers	NG	NG	Denmark	Europe	EURO	NG	NG	High	NG	NG	NG	NG	NG	NG	NG	5958	15036	NG	NG	858	NG	NG
Sahebi et al,2010	2010	542	542	Night workers	NG	NG	Iran	Asia	EMRO	NG	NG	High	23	23	NG	NG	NG	NG	NG	NG	NG	NG	NG	NG	NG	NG
Urbanetto et al,2015	2015	388	188	Night workers	73	315	Brasil	America	AMRO	NG	NG	High	61	61	NG	NG	NG	260	260	82	237	203	NG	NG	NG	NG
Saberinia et al,2018	2018	250	250	Shift workers	NG	NG	Iran	Asia	EMRO	NG	NG	High	11	13	NG	NG	28	NG	NG	59	NG	NG	NG	NG	NG	NG
Miller et al,2007	2007	749	749	Shift workers	NG	NG	United States	America	AMRO	NG	NG	High	NG	NG	NG	NG	NG	227	177	NG	NG	NG	NG	NG	NG	NG
Bahadar Khan et al,2012	2012	165	165	Shift workers	11	154	Pakistan	Asia	EMRO	40.75	8.57	High	31	31	NG	NG	4	NG	NG	1	104	NG	18	2	55	NG
Jahromi et al,2017	2017	263	263	Day workers	53	210	Iran	Asia	EMRO	31.04	6.97	High	24	20	25	16	14	51	51	NG	NG	NG	7	NG	NG	NG
