# Supplementary material for: Cardiovascular risk factors among nurses: A global systematic review and meta-analysis
Source: PLoS One. 2024 Mar 21;19(3):e0286245. doi: 10.1371/journal.pone.0286245 (PMC10956831; doi:10.1371/journal.pone.0286245)
Supplement: Attachment — Submitted filename: Dear editor-comments-CVD.docx [file pone.0286245.s003.docx]

Dear editor,

First of all, we appreciate the reviewers' comments regarding the manuscript "Cardiovascular risk factors among nurses: A global systematic review and meta-analysis”. We went through all the comments carefully and revised the necessary parts as highlighted in green. Furthermore, to clarify the applied changes we replied to each of the reviewers' comments as below.

Reviewer: 1

**Comment 1:** The manuscript is technically sound and data support the conclusions.

**Response to comment 1:** We appreciate the positive attitude of the reviewer toward the manuscript.

**Comment 2:** The statistical analysis has been performed appropriately.

**Response to comment 2:** We appreciate the positive attitude of the reviewer toward the statistical analysis.

**Comment 3:** All data underlying the findings should be fully available in the supplementary file.

**Response to comment 3:** We provided all data underlying the findings in the supplementary file.

**Comment 4:** The manuscript is presented in an intelligible fashion.
**Response to comment 4:** We appreciate the positive attitude of the reviewer toward the manuscript presentation fashion.

**Comment 5:** The introduction is good but it is better to add some references in the field.

**Response to comment 5:** We added all suggested references in the introduction section.
**Comment 6:** The review protocol should have been pre-registered in PROSPERO.
**Response to comment 6:** We pre-registered the review protocol and relevant information was added in to methodology section. This SLR has been registered on PROSPRO with code CRD42022329794

**Comment 7:** Provide the full electronic search strategies for databases in a supplementary file.

**Response to comment 7:** We added the required information in Method section.

**Comment 8:** Data screening and selection should have Kappa statistics between the authors.

**Response to comment 8:** We added Kappa statistics.

**Comment 9:** Include a copy of PRISMA checklist as a supplementary appendix.

**Response to comment 9:** We included a copy of PRISMA checklist in a supplementary appendix.

**Comment 10:** Results were duplicated in the text and tables/ figures.

**Response to comment 10:** We avoided any duplication to improve the readability of the manuscript. We only reported the main findings of the study in the text.

**Comment 11:** More explanation of the results should be added to the discussion.

**Response to comment 11:** More explanation of results was added to the discussion.

Reviewer: 2

**Comment 1:** Add objective to the end of introduction.

**Response to comment 1:** We added objective to the end of introduction.

**Comment 2:** Add inclusion and exclusion criteria for the review.

**Response to comment 2:** The inclusion and exclusion of the review are highlighted in green in the methodology section.

**Comment 3:** The Newcastle-Ottawa scale for quality assessment should be stated in the method.

**Response to comment 3:** The Newcastle-Ottawa scale for quality assessment was explained in the method and highlighted in green.

**Comment 4:** The results for family history of cardiovascular disease, being overweight and alcohol consumption should be present since they are among the most prevalent risk factors in the study

**Response to comment 4:** We added the mentioned factors as most prevalent risk factors in the study.

**Comment 5:** Regarding systemic blood pressure, the authors should present the prevalence of systemic arterial hypertension instead of the mean systolic and diastolic blood pressures.

**Response to comment 5:** This was one of the goals of our research, but, unfortunately these type of data did not exist on the included articles

**Comment 6:** Provide a summary of limitations of the evidence included in the review.

**Response to comment 6:** We provided a summary of limitations at the end of discussion section.

**Comment 7:** Present the main conclusions related to the study objective.

**Response to comment 7:** The main conclusions were presented regarding the study objective.

**Comment 8:** How the current systematic review can add to the acknowledgment from the previous studies.

**Response to comment 8:** We highlighted the explanation about what this systematic review adds to previous studies (in the last paragraph of introduction).

**Comment 9:** In the last paragraph, the authors should provide a more explicit statement of the objectives being addressed.

**Response to comment 9:** We stated the study objective in the last paragraph of introduction.

**Comment 10:** The literature review search period and the last search date should be stated in the first paragraph of the Methods.

**Response to comment 10:** The required information (The search was carried out from the beginning of the year 2000 to the end of March 2022) is highlighted in the methodology.

**Comment 11:** Data bases and search terms should be splited in to separate headings of study design and registration, search strategies, data collection and analysis, and data extraction and management.

**Response to comment 11:** The methodology section was splited based on the reviewer’s comment.

**Comment 12:** The authors should consider showing the complete search strategies for all databases, including any filters and limits used, in the Supplementary Material.

**Response to comment 12:** We provided the required data in the Method section.

**Comment 13:** The authors should consider the inclusion and exclusion criteria using the study characteristics and report characteristics.

**Response to comment 13:** We considered the inclusion and exclusion criteria in the methodology section as highlighted in green.

**Comment 14:** The working definition for behavioral, biological, and physical risk factors should be presented in the Methods.

**Response to comment 14:** As we searched for cardiovascular risk factors as a whole search term, we did not divide our searching process in to behavioral, biological and physical risk factors. Thus, in the result section we reported all identified risk factors all together irrespective of their category.

**Comment 15:** Why did the authors include only articles in English?

**Response to comment 15:** We only searched for articles in English and we regarded this as a study limitation.

**Comment 16:** The study selection process should be described in more detail: the duplicate screening, title and abstract screening, and full-text articles assessment for relevance. Did a reference management software package or a screening and data extraction tool be used to screen for duplicates and study selection?

**Response to comment 16:** The title/ abstract screening, full-text assessment, and a reference management software package used to screen for duplicates were mentioned in the methodology section.

**Comment 17:** The risk of bias assessment should be stated in the Methods.

**Response to comment 17:** The risk of bias assessment was stated in the methodology section (identified by NOS tool).

**Comment 18:** The number of studies excluded for each criterion should be shown in Figure 1.

**Response to comment 18:** The number of studies excluded for each criterion was shown in Figure 1.

**Comment 19:** In the text, before splitting into behavioral, biological, and physical risk factors, the authors could present the results of all cardiovascular risks together.

**Response to comment 19:** We presented the results of all cardiovascular risks together as presented in Table 2.

**Comment 20:** The authors should present the results of the meta-analysis as forest plots, including the heterogeneity test (I^2^).

**Response to comment 20:** Based on the data and type of analysis, Table was preffered rather than forest plot.

**Comment 21:** Please correct the p-values that cannot be 0.000 (insert p < 0.001 instead p = 0.000).

**Response to comment 21:** We made necessary corrections.

**Comment 22:** The full form of abbreviations should be presented in the footnote.

**Response to comment 22:** We made necessary changes.
