# Supplementary material for: Cardiovascular risk factors among nurses: A global systematic review and meta-analysis
Source: PLoS One. 2024 Mar 21;19(3):e0286245. doi: 10.1371/journal.pone.0286245 (PMC10956831; doi:10.1371/journal.pone.0286245)
Supplement: Attachment — Submitted filename: Dear editor-comments-CVD.docx [file pone.0286245.s004.docx]

Dear editor,

First of all, we appreciate the reviewers' comments regarding the manuscript "Cardiovascular risk factors among nurses: A global systematic review and meta-analysis”. We went through all the comments carefully and revised the necessary parts as highlighted in green. Furthermore, to clarify the applied changes we replied to each of the reviewers' comments as below.

Reviewer: 1

**Comment 1:** The key inclusion and exclusion criteria for the review and language restrictions should be stated in the Abstract Methods.

**Response to comment 1:** from the beginning of 2000 to March 2022

**Comment 2:** The Newcastle-Ottawa Scale for quality assessment and the risk of bias assessment should be stated in the Abstract Method.

**Response to comment 2:** the Newcastle-Ottawa Scale (NOS) was used to assess the methodological quality of included studies.

**Comment 3:** Regarding the risk factors, family history of cardiovascular disease (41.9%; 95% IC: 29.8-55.1%), being overweight (33.3%; 95% IC: 24.7-43.2%), and alcohol consumption (24.6%; 95% IC: 16.4-35.2%) should be present in the Abstract Results since they are among the most prevalent in the study.

**Response to comment 3:** Also family history of cardiovascular disease (41.9%; 95% IC: 29.8-55.1%), being overweight (33.3%; 95% IC: 24.7-43.2%), and alcohol consumption (24.6%; 95% IC: 16.4-35.2%) was found among the participants.

**Comment 4:** The authors should summarize the limitations of the evidence included in the review, such as the quality of the evidence and the risk of bias, inconsistency, and imprecision in the Abstract Results.

**Response to comment 4:** By adding this section to the abstract, it would exceeded the limitation word.
